# *MicroRNA319* Positively Regulates Cold Tolerance by Targeting *OsPCF6* and *OsTCP21* in Rice (*Oryza sativa* L.)

**DOI:** 10.1371/journal.pone.0091357

**Published:** 2014-03-25

**Authors:** Sun-ting Wang, Xiao-li Sun, Yoichiro Hoshino, Yang Yu, Bei Jia, Zhong-wen Sun, Ming-zhe Sun, Xiang-bo Duan, Yan-ming Zhu

**Affiliations:** 1 Key Laboratory of Agricultural Biological Functional Genes, Northeast Agricultural University, Harbin, P.R. China; 2 Field Science Center for Northern Biosphere, Hokkaido University, Kita-ku, Sapporo, Hokkaido, Japan; Cankiri Karatekin University, Turkey

## Abstract

The microRNA319 (miR319) family is conserved among diverse plant species. In rice (*Oryza sativa* L.), the miR319 gene family is comprised of two members, *Osa-miR319a* and *Osa-miR319b*. We found that overexpressing *Osa-miR319b* in rice resulted in wider leaf blades and delayed development. Here, we focused on the biological function and potential molecular mechanism of the *Osa-miR319b* gene in response to cold stress in rice. The expression of *Osa-miR319b* was down-regulated by cold stress, and the overexpression of *Osa-miR319b* led to an enhanced tolerance to cold stress, as evidenced by higher survival rates and proline content. Also, the expression of a handful of cold stress responsive genes, such as *DREB1A/B/C*, *DREB2A*, *TPP1/2*, was increased in *Osa-miR319b* transgenic lines. Furthermore, we demonstrated the nuclear localization of the transcription factors, *OsPCF6* and *OsTCP21*, which may be *Osa-miR319b*-targeted genes. We also showed that *OsPCF6* and *OsTCP21* expression was largely induced by cold stress, and the degree of induction was obviously repressed in plants overexpressing *Osa-miR319b*. As expected, the down-regulation of *OsPCF6* and *OsTCP21* resulted in enhanced tolerance to cold stress, partially by modifying active oxygen scavenging. Taken together, our findings suggest that *Osa-miR319b* plays an important role in plant response to cold stress, maybe by targeting *OsPCF6* and *OsTCP21*.

## Introduction

Rice is a kind of important food and economic crop, and its production is frequently affected by cold stress. Cold stress is one of the most important limiting factors affecting rice production in temperate and high elevation areas [1]. During the early growth stages, cold stress severely inhibits rice seedling establishment and eventually leads to non-uniform maturation [2]. Cold stress always imposes a series of negative effects on photosynthesis, respiration, and the production and accumulation of reactive oxygen species (ROS) [3], [4]. However, rice has evolved efficient mechanisms to rapidly sense and respond to cold stress. To understand the underlying molecular mechanisms of the cold stress response, many intensive studies have been performed. MicroRNAs (miRNAs) are small single-stranded non-coding RNAs, usually ∼21 bp in length, which regulate biological processes through mediating translational inhibition or cleavage of target transcripts [5], [6], [7], [8]. Several studies have reported the involvement of miRNAs and their target genes in plant responses to stress conditions [9], [10], [11]. Under cold stress, the up- or down-regulation of miRNAs modifies the transcript abundance of their target genes [12], [13].

The *miR319* family is one of the most ancient and conserved miRNA families, and has been found in a large number of plant species, from *Physcomitrella* to flowering plants [14], [15], [16], [17], [18], [19]. *MiR319*, previously known as ‘miR-JAW’, was firstly described in *Arabidopsis* because of its role in controlling leaf morphogenesis [20], [21]. It has been reported that miR319 targeted TEOSINTE BRANCHED/CYCLOIDEA/PCF (TCP) genes, the plant-specific transcription factors containing bHLH motifs that allow DNA binding and protein-protein interaction [22], [23], [24], [25], [26], [27]. Microarray data of the shoot apical meristem of *miR319* transgenic plants, compared to wild-type (WT), showed that expression of all *miR319*-targeted TCP genes decreased up to 30-fold, which strongly indicated that TCP genes were degraded by *miR319* activity [20], [28], [24]. Expression of TCPs, lacking the *miR319* recognition site were not affected. Previous studies showed that *miR319*-TCPs regulated leaf developmental processes, including leaf senescence, cell proliferation, and cell differentiation [20], [29], [30].

In addition, several researchers have suggested the involvement of *miR319* in the cold stress response. In sugarcane, changes in *miR319* expression under cold stress were observed in both roots and shoots [31]. In rice, expression of *Osa-miR319* was down-regulated by cold stress [13]. Yang et al. (2013) observed that overexpression of *Osa-miR319b* and repression of its targets, *OsPCF5* and *OsPCF8* led to enhanced cold tolerance (4°C) after chilling acclimation (12°C). *MiR319* was supposed to participate in plant cold tolerance by regulating its target genes or functioning together with other miRNAs [27]. However, little is known about the regulatory relationship between *miR319* and its targets, *OsPCF6* and *OsTCP21*, under cold stress.

In previous studies, we constructed a rice miRNA expression profile under cold stress based on the microarray data, and identified a total of 18 cold stress responsive miRNAs, including *Osa-miR319*. In this study, we further characterized the biological function of *Osa-miR319* in response to cold stress by using overexpression transgenic lines. To elucidate the regulatory relationship between *OsmiR319* and *OsPCF6*/*OsTCP21*, we evaluated the effect of *Osa-miR319* overexpression on *OsPCF6* and *OsTCP21* expression under cold stress. We also investigated the roles of *OsPCF6* and *OsTCP21* in plant cold response and ROS accumulation under cold stress by using overexpression and RNAi transgenic rice. Collectively, the results suggested that, as *Osa-miR319* targets, *OsPCF6* and *OsTCP21* negatively regulated plant cold tolerance.

## Materials and Methods

### Plant materials and stress treatments

De-hulled seeds of *Oryza sativa* L. (Kong Yu 131, from the College of Agriculture, Northeast Agricultural University, Harbin, Heilongjiang Province, China) were sterilized with 5% sodium hypochlorite (NaClO) for 15 min and rinsed with sterilized water for three times. The sterilized seeds were germinated on 1/2 Murashige and Skoog (MS) medium with 1% (W/V) agar for 4 d, and then transferred to Yoshida nutrient solution under normal culture conditions (25–28°C, 600 µmol photons m^−2^ s^−1^, 14 h light/10 h dark cycles, 75% relative humidity) [52]. For gene expression analysis, the 2-week-old seedlings were exposed to 4°C, and equal amounts of leaves were harvested at certain time points. After being frozen in liquid nitrogen, the samples were stored at −80°C before RNA extraction.

### RNA isolation, cDNA synthesis and real-time RT-PCR analysis

Total RNA was extracted by using a plant RNA isolation reagent (Cat. No. 12322-012, Invitrogen, Carlsbad, CA, USA) following the manufacturer's protocol. Reverse transcription reactions were performed by using M-MLV reverse transcriptase (Cat. No. M0253V, NEB, Ipswich, MA, USA). *OsEf1-α* (elongation factor 1-α) was used as an internal control. The 2^−ΔΔCt^ method was used for real-time PCR analysis. To enable statistical analysis, three fully independent biological replicates were obtained and subjected to real-time PCR in triplicate. Primers for quantitative real-time PCR are listed in Table 1.

**Table 1:** Primers of genes used for quantitative real-time PCR validation.

Gene	Prime sequence
***Pre-OsR319b***	Forward: 5′- GTCGAATTAGCTGCCGACTC -3′
	Reverse: 5′-TCGAAGAGATCGAGGAGGAG-3′
***OE-OsPCF6***	Forward: 5′-GTGCCAATAGGGGGACCCT-3′
	Reverse: 5′-CCAAGCAGGAAGGAAATGGT-3′
***OE-OsTCP21***	Forward: 5′-CACTTCATGCCCGTCCACG-3′
	Reverse: 5′-TGGCTGGAGAGGTGAGACTGC-3′
***Ri-OsPCF6***	Forward: 5′-ATCGGTTGCCTGTCAATTTT-3′
	Reverse: 5′-GATTTATCTCAACTTTATAG-3′
***Ri-OsTCP21***	Forward: 5′-TTTTTGGCCATCATGAACAC-3′
	Reverse: 5′-GATTTATCTCAACTTTATAG-3′
***J033099M14***	Forward:5′-CTCAAATCAAGGCGTCAACTAAGA-3′
	Forward:5′-TTTGTCAATATATACGTGGCATATACCA-3′
***OsSIK1***	Forward:5′-TCTGGTAGTCTGCCCGAGGAA-3′
	Forward:5′-TATGTACTGGTTGCAATCAG-3′
***Os01g22249***	Forward:5′-AACGGAGTGGAAGCAGCGT-3′
	Forward:5′-CAGCACCTCTATGTTGCCCA-3′
***DREB1A***	Forward:5′-GGAGCAAGCAGAAACACACA-3′
	Forward:5′-TCGTCTCCCTGAACTTGGTC-3′
***DREB2A***	Forward:5′-AAGCACGGCATAATTTTTGG-3′
	Forward:5′-CGTTCTTTCCAGCTTTCCTG-3′
***DREB1B***	Forward:5′-CTCGCACTGAAAAGTGTGGA-3′
	Forward:5′-CAAAAGGAGGGAGAAATCTGG-3′
***DREB1C***	Forward:5′-CTACGCGTACTACGGCAACA-3′
	Forward:5′-GAGGAGCAAAGCTGGTTGAG-3′
***Tpp1***	Forward:5′-CCTTCAGCAAATCATGAGCA-3′
	Forward:5′-AGCCTCCAGCACTTCGTTTA-3′
***Tpp2***	Forward:5′-AGGATGCATTCAAGGTTCTGA-3′
	Forward:5′ -CAAGATGCCAGTTTCTTCAGG-3′
***OsCPT1***	Forward:5′-AGTCGCGTGTTCTCCTTTGT-3′
	Forward:5′-CATGACAGCAGCTTGCAAAT-3′

### Vector construction and generation of transgenic rice

*Osa-miR319b* pre-sequences were downloaded from miRBase (http://www.mirbase.org) and PCR amplified using gene specific primers (5′-GGCTTAAUGCTCCAAAAGTTTCGTGGTTGTT-3′ and 5′-GGTTTAAUACTGGTGCTATCATTTCATGCCC-3′). The PCR products were then cloned into the binary vector pCAMBIA330035sU to create the *Osa-miR319b* overexpression construct driven by the CaMV35s promoter [60]. The full-length coding region of *OsPCF6* was amplified with gene specific primers 5′-GAGCCTCTCTCGCGGAACAAGGCAT-3′ and 5′-GAGGATGCGTTGCTGGGATCGATCA-3′; the *OsTCP21* gene was amplified using the specific primers 5′-TCCTAGGGCATATGACCGGTACTACG-3′ and 5′-GCTGCTGATCTGATCAGTGCTTGCC-3′. The PCR products were ligated into the pBluescript SK vector (TransGen Biotech Beijing China) for sequence, and then, digested by *BamH*I and *Xba*I, and cloned into pCAMBIA3300 to obtain the pCAMBIA3300-*OsPCF6* and pCAMBIA3300-*OsTCP21* constructs. To generate the RNA interference (RNAi) constructs, *OsPCF6* and *OsTCP21* were amplified using specific primers, *OsPCF6*: 5′-ATCGGTTGCCTGTCAATTTT-3′ and 5′-GATTTATCTCAACTTTATAG-3′; and *OsTCP21*: 5′-TTTTTGGCCATCATGAACAC-3′ and 5′-GATTTATCTCAACTTTATAG-3′, respectively, and cloned into pCAMBIA3300 by forward and reverse insertions. The recombinant constructs were introduced into *Agrobacterium tumefaciens* EHA105 and then transformed into rice embryonic calli. The re-generated seedlings were selected on 1/2 MS medium containing 15 mg L^−1^ glufosinate (Roche, Germany).

The presence of the *Osa-miR319b*, *OsPCF6* or *OsTCP21* genes in the glufosinate-positive plants were confirmed by PCR analysis using CaMV35S promoter-specific forward primer and the *Bar* gene-specific reverse primer. The PCR-positive seedlings were further confirmed by southern blot analysis. Briefly, genomic DNA extracted from 14-day-old seedlings was digested with *Hind*III, electrophoresed on a 0.8% (W/V) agar gel, and blotted onto a nylon membrane (Roche, Germany) under alkaline conditions. Digoxingenin-dUTP amplified *Bar* from pCAMBIA330035sU was used as a probe for hybridization. The membrane was exposed to X-ray film (Eastern Kodak). Transcript levels of *Osa-miR319b*, *OsPCF6* and *OsTCP21* genes in transgenic rice were analyzed by using semi-quantitative RT-PCR or quantitative real-time PCR analysis using gene specific primers. The *OsEf1-α* (elongation factor 1-α) was used as an internal control.

### Phenotypic analysis of transgenic rice under cold stress

For the cold tolerance test, three-leaf seedlings of both WT and transgenic lines were exposed to 4°C for 7 d, and then recovered under normal conditions for 10 d. Survival rate of WT and transgenic plants lines after 10 days of recovery. The proline content in rice leaves was determined by the method described previously [53], [54].

DAB is oxidized by hydrogen peroxide in the presence of some heme-containing proteins, such as peroxidases, to generate a dark brown precipitate [55]. DAB staining of the rice aerial parts were determined according to the method described previously [56], [57], [58].

### Subcellular localization of the OsPCF6 and OsTCP21-GFP fusion protein

To determine the subcellular localization, the full-length *OsPCF6* and *OsTCP21* coding regions were inserted into the pBSK:eGFP vector to generate pBSK-OsPCF6-GFP and pBSK-OsTCP21-GFP. The constructs were confirmed by sequencing and used for transient transformation of onion (*Allium cepa*) epidermal cells by biolistic bombardment. After 24 h of incubation, GFP fluorescence in transformed onion cells was observed at 488 nm with a confocal microscope (Zeiss, Germany). GFP fluorescence was detected between 505 nm and 550 nm with an excitation wavelength of 488 nm. GFP fluorescence and light field visions were recorded in separate channels and then merged into an overlay image.

### Statistics

All numerical data was analyzed using the Student's t-test. Statistical differences were referred as significant when *P<0.05 and **P<0.01.

## Results

### Production and molecular characterization of *Osa-miR319b* transgenic *Oryza sativa* plants

**Figure 1:**
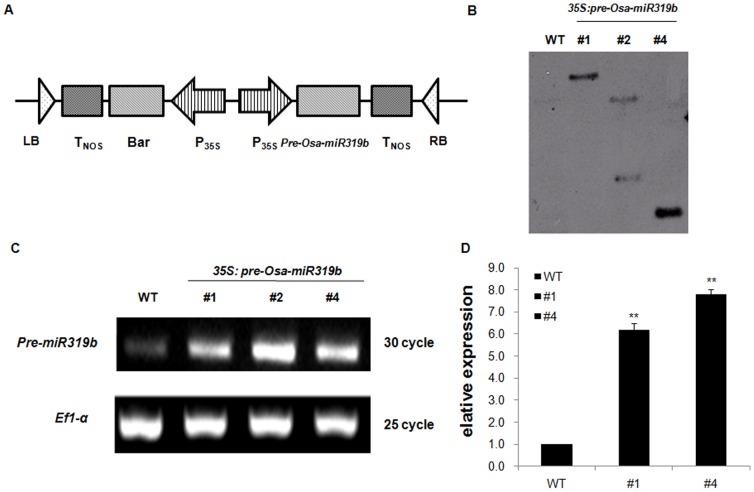
Generation and molecular analysis of *Osa-miR319b* transgenic lines. (A) Schematic diagram of the *Osa-miR319b* overexpression gene construct, p35S-*Osa-miR319b*/p35S-*Bar*, in which the *Osa-miR319b* gene is under the control of the CaMV 35S promoter. The CaMV 35S promoter-driven *Bar* is included for glufosinate resistance. LB, Left border; RB, right border. (B) Southern blot analysis of transgenic rice. Rice DNA and vector DNA were digested by HindIII, run on a 0.8% agarose gel, transferred to a nylon membrane, and probed with digoxingenin-dUTP against the amplified bar gene sequence. The wild-type rice DNA was used as negative control. (C) Expression pattern of *Osa-miR319b* in wild-type and transgenic lines by semi-quantitaive RT-PCR. cDNAs were normalized using the *Ef1-α* gene. (D) Verification of the mature-*Osa-miR319b* overexpression in *Osa-miR319b*-overexpressing transgenic rice lines by real-time RT-PCR. RNA was extracted from the flag leaves of the second generation of the transgenic rice plants. snRNA U6 served as a reference gene for the detection of miRNAs.

In previous studies, we identified a total of 18 cold stress responsive miRNAs using microarray data, including miR-156k, miR-166k, miR-166m, miR-167a/b/c, miR-168b, miR-169e, miR-169f, miR-169h, miR-171a, miR-535, miR-319a/b, miR-1884b, miR-444a.1, miR-1850, miR-1868, miR-1320, miR-1435, and miR-1876 [13]. In the current study, we focused on the *Osa-m*iR319b gene, which was obviously down-regulated under cold stress. To explore the potentiality of manipulating *Osa-miR319b* expression in annual species for enhancing plant resistance to cold stress, we prepared a chimeric construct, p35S-*Osa-miR319b*/p35S-*Bar*, under the control of the CaMV 35S promoter (Fig. 1A). The transgenic lines were identified by glufosinate selection, PCR and southern blot analysis. Southern blotting results showed that *Osa-miR319b* was integrated into the rice genome of three independent transgenic lines (Fig. 1B). Semi-quantitative RT-PCR analysis showed relatively higher expression levels of *Osa-miR319b* in all three lines (Fig. 1C). Expression levels of the mature-*Osa-miR319b* in transgenic lines were further confirmed by quantitative real-time PCR analysis (Fig. 1D). The homozygous T_2_ generation of two single-copy transgenic lines (#1 and #4) was used in the following experiments.

### Overexpression of *Osa-miR319b* in rice results in increased leaf width

**Figure 2:**
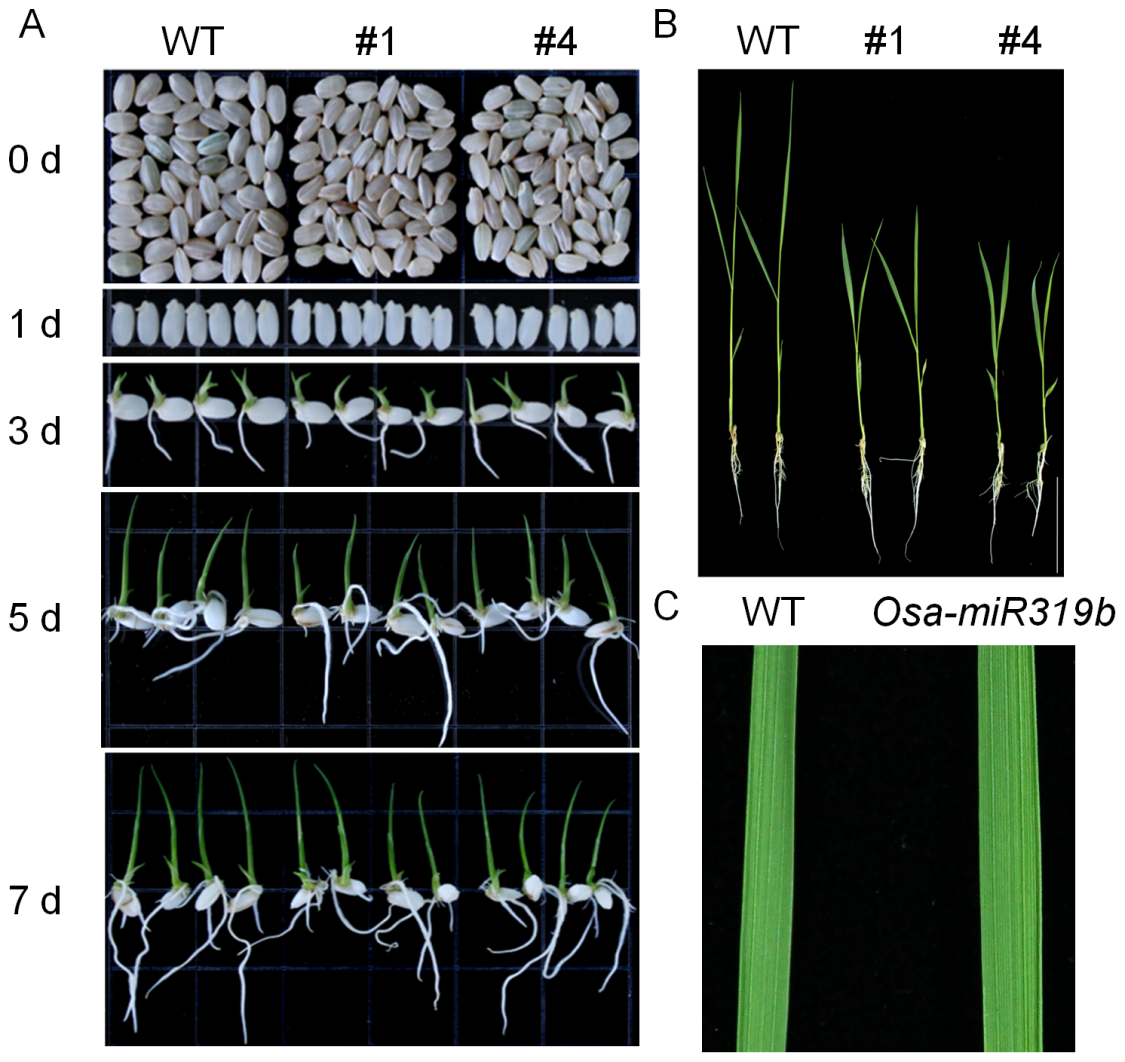
*Osa-miR319b* overexpressing plants showed delayed development and widen leaf. (A) Gross morphology at 0 to 7-day-old seedlings phase of WT and transgenic plants; (B) Transgenic plants showed 14 d delaying of heading time comparing with WT. (C) The *Osa-miR319b*-overexpressing transgenic plants exhibited wider leaves than WT controls.

The most conspicuous feature of *Osa-miR319b*-overexpressing transgenic plants (OE) is delayed development and wider leaves. It is noteworthy that the size of OE seed is smaller than wild-type seed. Gross morphology at 1–5 day old seeding phase of wild-type and transgenic plants have no remarkable different. However, gross morphology of transgenic plants are smaller than wild-type plants at two-week-old (Fig. 2B), but the leaf blade of transgenic plant is larger compared with wild-type blade (Fig. 2C). There is no obviously difference in plant height and the number of productive tillers per plant at final period (data not shown). These results indicated that overexpression of *Osa-miR319b* possibly conferred plants developments.

### Overexpression of *Osa-miR319b* in *O.sativa* enhance plant tolerance to cold stress

**Figure 3:**
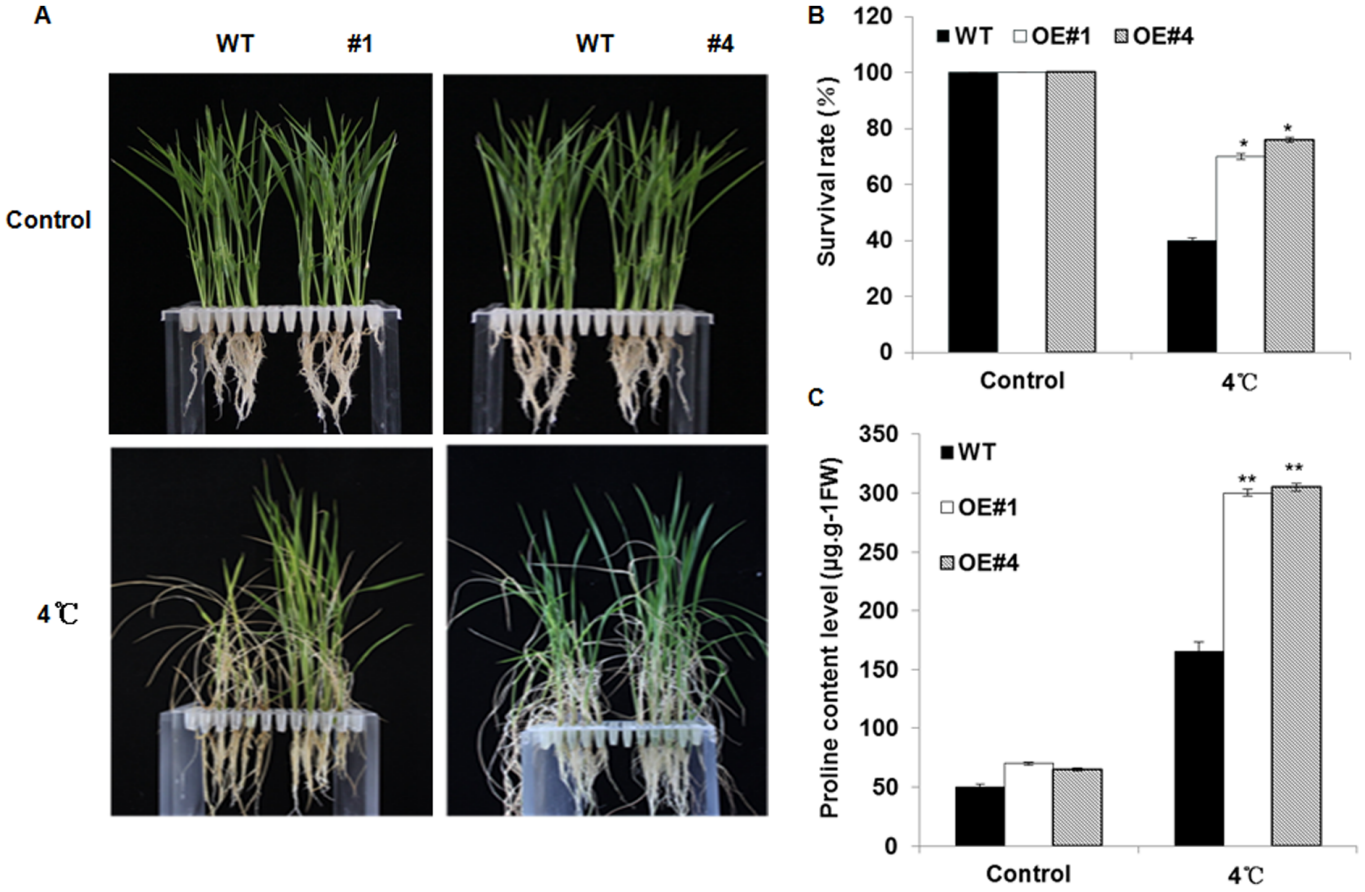
Stress tolerance of *Osa-miR319b* transgenic rice and wild-type rice subjected to cold stress treatment. (A) Growth performance of WT and transgenic rice (#1 and #4) under normal condition and cold stress. The WT and transgenic rice seedlings were exposed to 4°C for 7 d followed by 10 d of recovery. (B) The survival rates of WT and transgenic seedlings under normal condition and cold stress. The numbers of survived seedlings/total seedlings used to calculate the survival rate. (C) The free proline content of WT and transgenic seedlings under normal condition and cold stress. Wild-type and transgenic rice seedlings of 2-week-old were exposed to 4°C for 3 days and then collected for determination of proline. Data are the mean ± SE of three biological replicates. Asterisks indicate statistically significant differences (*P<0.05, **P<0.01) between wild-type and transgenic lines.

To test whether *Osa-miR319b* overexpression affects plant tolerance to cold stress, the WT and overexpression (OE) seedlings were hydroponically cultured in Yoshida solution until three leaves appeared. The seedlings were directly exposed to 4°C for 7 d, and then recovered for 10 d. As shown in Figure 3A, the OE lines showed significantly better growth performances than WT. The statistical analysis revealed that the survival rate of WT seedlings under cold stress was approximately 30%, while OE lines #1 and #4 displayed approximately 80% and 90% survival rates respectively (Fig. 3B) (P<0.05, by Student's t-test). These results demonstrated that *Osa-miR319b* overexpression enhanced plant cold tolerance at the seedling stage.

The accumulation of proline to facilitate osmoregulation is a common adaptive mechanism for plant tolerance to cold stress. To test whether the enhanced cold tolerance of *Osa-miR319b* OE plants is related to the capacity of proline accumulation, the free proline contents of WT and OE plants were investigated. As shown in Figure 3C, no significant difference was observed between WT and OE plants under normal conditions. Cold stress obviously promoted the free proline accumulation in both WT and OE plants. However, the proline content of OE plants was significantly higher than that of WT (Fig. 3C) (P<0.01, by Student's t-test). These results indicated that overexpression of *Osa-miR319b* conferred increased cold tolerance, perhaps through promoting the free proline accumulation under cold stress.

### Overexpression of *Osa-miR319b* increases the transcript levels of cold stress responsive genes

**Figure 4:**
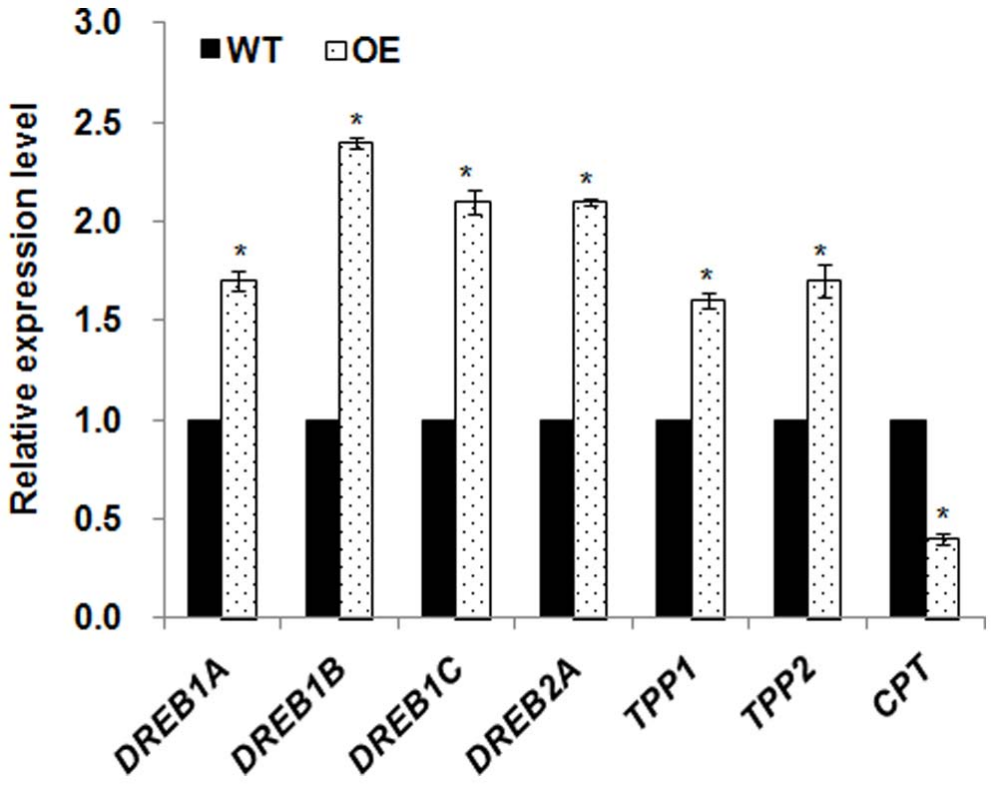
Expression levels of some cold-responsive genes in wild-type plants and overexpressing *Osa-miR319b* plants. Marker gene expression levels were set equal to 1 in the WT plants in cold stress with 4°C for 8 h. The expression level of marker genes are represented as a ratio in OE plants compared with WT plants. Data are the mean ± SE of three biological replicates. Asterisks indicate statistically significant differences (*P<0.05) between WT and transgenic lines.

To explore the role of *Osa-miR319b* in the cold stress response, the expression of the well-known cold stress responsive marker genes was analyzed in both WT and OE plants. Marker genes linked to cold stress, such as *DREB1/CBF, DREB2A, OsCPT1, OsTPP1/2*, could contribute to plant cold enhanced tolerance. To test whether these genes were responsible for enhanced the cold tolerance of *Osa-miR319b* OE plants, quantitative real-time PCR was performed to evaluate the expression levels. As shown in Figure 4, cold stress obviously induced expression of all the above genes, and *Osa-miR319b* OE lines exhibited significantly higher expression levels than WT, except OsCPT (P<0.05, by Student's t-test). *OsCPT* is a putative target member of the dehydration-responsive element-binding factor 1 DREB1/CBF pathway, and showed opposite expression to DREB1/CBF under cold stress. Thus, *Osa-miR319b* may confer increased cold tolerance through up-regulating cold stress responsive genes, including *DREB1/CBF*, *DREB2A*, and *TPP1/2*.

### The cold induction of *OsPCF6* and *OsTCP21* is obviously repressed in *Osa-miR319b* OE lines

**Figure 5:**
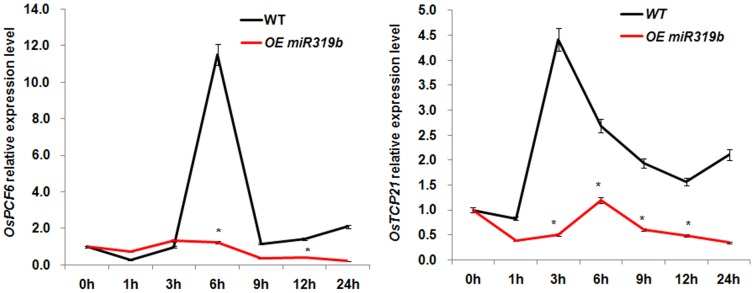
qRT-PCR analysis of *OsPCF6* and *OsTCP21* expression patterns under 4°C in wild-type and *Osa-miR319b*-overexpressing plants. Expression patterns of the *OsPCF6* and *OsTCP21* genes under 4°C in rice seedlings at 0, 1, 3, 6, 9, 12, 24 h after cold stress onset in WT and *Osa-miR319b*-overexpressing lines. The expression level at 0 h was set at 1.0. Data are the mean ± SE of three biological replicates. Asterisks indicate statistically significant differences (*P<0.05) between wild-type and transgenic lines.

In previous studies, *PCF6* gene was identified as one of the *miR319b* targets by using 5′RACE analysis in sugarcane [32]. Through Blastp search, we identified two homologous genes *OsPCF6* and *OsTCP21*. Thus, we analyzed the expression profiles of *OsPCF6* and *OsTCP21* in WT and *Osa-miR319b* OE lines in response to cold stress, to verify whether their expression was affected by *Osa-miR319b* overexpression. The quantitative real-time PCR results showed that expression of *OsPCF6* and *OsTCP21* was significantly induced by cold stress in WT plants (Fig. 5). However, in the *Osa-miR319b* OE lines, the cold induction of *OsPCF6* and *OsTCP21* was obviously inhibited (P<0.05, by Student's t-test), which indicated that *Osa-miR319b* overexpression repressed the induced expression of *OsPCF6* and *OsTCP21* under cold stress. These results suggested that *OsPCF6* and *OsTCP21* were indeed target genes of *Osa-miR319b*, and were possibly involved in plant response to cold stress.

### *OsPCF6* and *OsTCP21* negatively regulates plant cold tolerance

**Figure 6:**
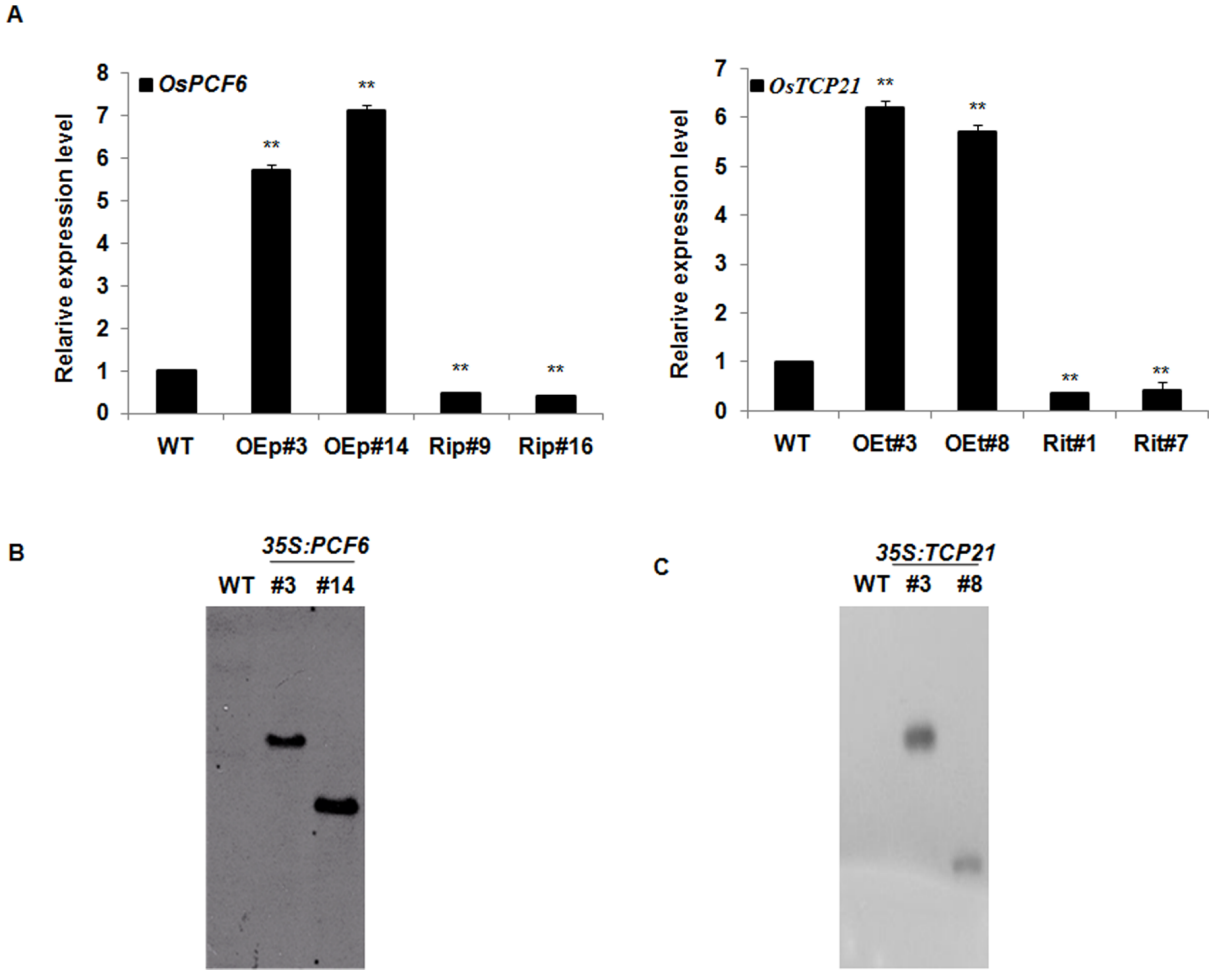
Molecular confirmation of *OsPCF6* and *OsTCP21* transgenic rice. (A) *OsPCF6* and *OsTCP21* expression in wild-type and transgenic rice. Total RNAs from 2-week-old wild-type and transgenic rice plants were isolated, reverse-transcribed, and analysed by real-time reverse-transcription PCR. cDNAs were normalized using the *Ef1-α* gene. Data are the means ± SE of three biological replicates. Asterisks indicate statistically significant differences (**P<0.01) between WT and transgenic lines (OE and Ri). (B) Southern blot analysis of *OsPCF6*. (C) Southern blot analysis of *OsTCP21*. The WT rice genomic DNA was used as negative control.

To test whether *OsPCF6* and *OsTCP21* were directly involved in plant response to cold stress, we generated OE and RNAi (Ri) transgenic lines, which were confirmed by glufosinate selection, quantitative real-time PCR (Fig. 6A) and southern blot analysis (Fig. 6B). Compared with WT, the transcript abundance of *OsPCF6* and *OsTCP21* was significantly higher in the *OsPCF6* and *OsTCP21* OE lines (OEp#3 and #14; OEt#3 and #8) but lower in the *OsPCF6* and *OsTCP21* Ri lines (Rip#9 and #18; Rit#1 and #7) (P<0.01, by Student's t-test). Seedlings of WT and homozygous T_2_ progeny were grown in Yoshida solution and used in the following phenotypic experiments. No differences were observed among WT, OE and Ri lines when grown under normal, non-stressed conditions.

**Figure 7:**
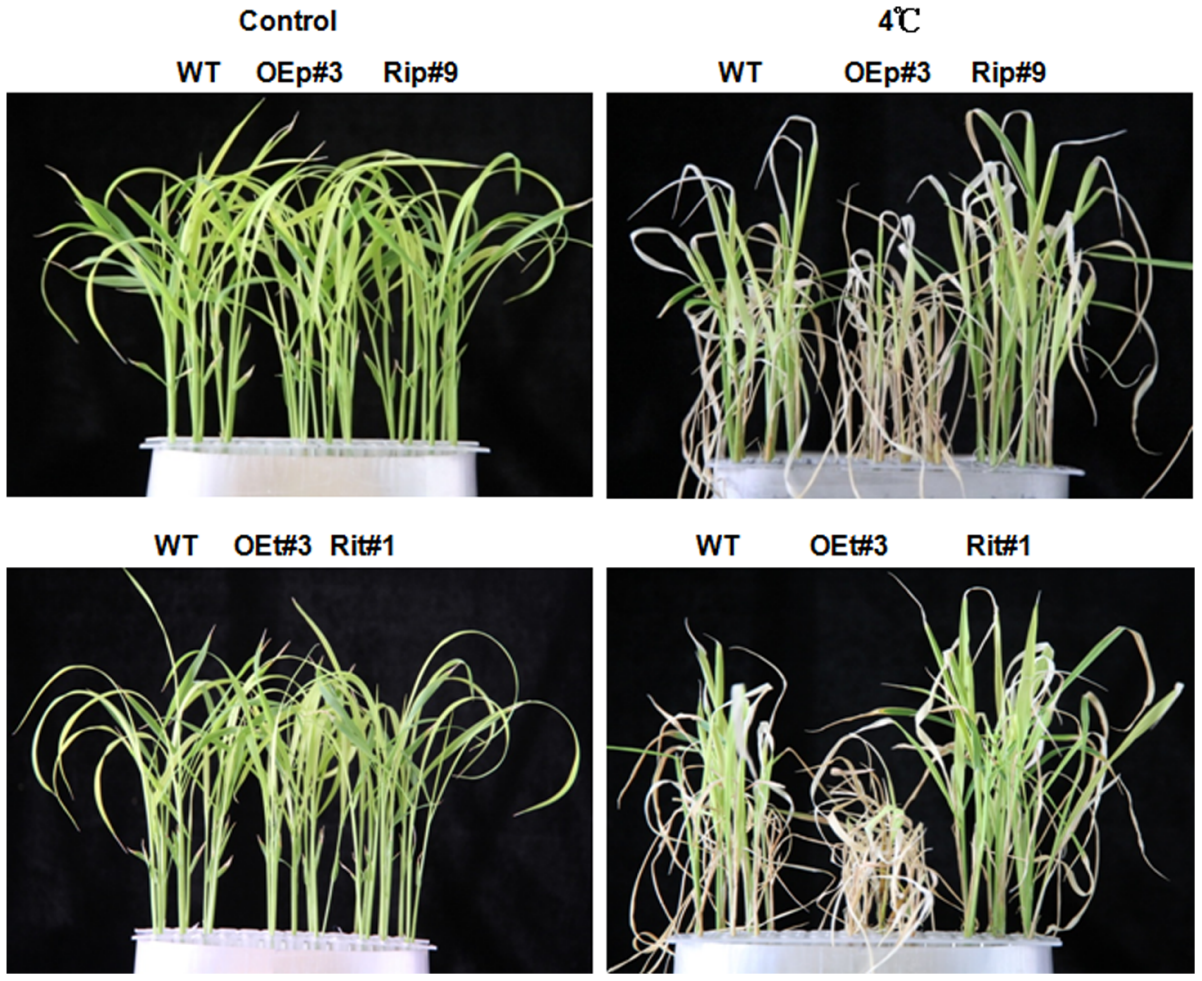
Effect of cold stress on *OsPCF6* and *OsTCP21* in wild-type and transgenic plants. Left: wild-type, *OsPCF6* and *OsTCP21* transgenic plants (OE and Ri) without stress treatment; right: wild-type and transgenic seedlings treated with 4°C for 7 d followed by 10 d of recovery.

**Figure 8:**
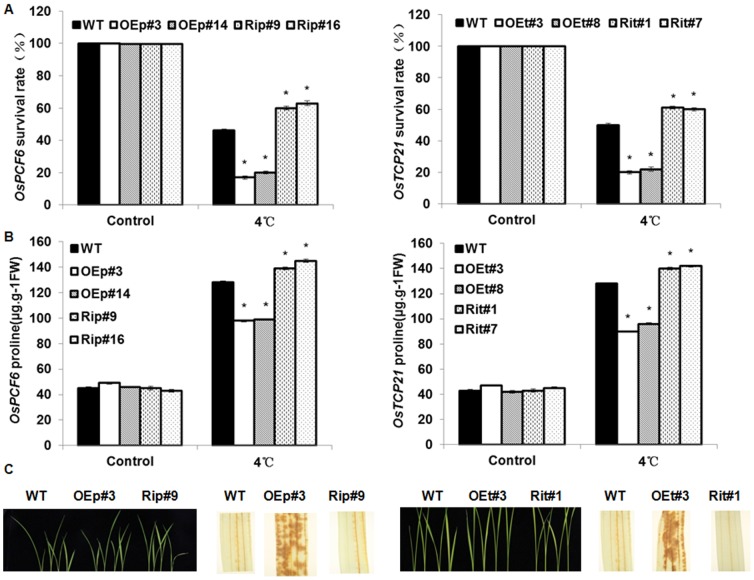
Transgenic plants (OE and Ri) of *OsPCF6* and *OsTCP21* confer cold tolerance in rice. (A) The survival rates of WT, *OsPCF6* and *OsTCP21* transgenic seedlings under normal condition and cold stress. (B) The proline contents of WT, *OsPCF6* and *OsTCP21* transgenic seedlings under normal condition and cold stress. Wild-type and transgenic rice seedlings of 2-week-old were exposed to 4°C for 3 days and then collected for determination of proline. (C) H_2_O_2_ accumulation in WT, *OsPCF6* and *OsTCP21* transgenic plants under cold stress. The 2-week-old seedling were exposed to 4°C for 3 d, and then seedling roots were cut off and directly incubated in the DAB dye solution for 8 h for H_2_O_2_ analysis. Left: WT, *OsPCF6* and *OsTCP21* transgenic plants without stress treatment; right: wild-type and transgenic plants, after treatment and staining. OE is dark brown; WT and Ri lines have the lightest color.

To investigate the involvement of *OsPCF6* and *OsTCP21* in cold stress, the WT, OE and Ri seedlings were exposed to 4°C for 7 d, after which they were recovered for 10 d. Phenotypically, most *OsPCF6* and *OsTCP21* Ri seedlings remained green and showed continuous growth, whereas both WT and OE seedlings showed severe leaf rolling and wilting after cold stress (Fig. 7). As shown in Figure 8A, the survival rates of the OE and/or Ri lines were significantly lower and/or higher, respectively, than that of WT when exposed to cold stress, indicating that the alteration of *OsPCF6* and *OsTCP21* expression levels altered plant cold tolerance.

In addition, we also evaluated the free proline accumulation in *OsPCF6* and *OsTCP21* OE and Ri transgenic lines under cold stress. As shown in Fig. 8B, there was no significant difference in the proline contents among WT, OE and Ri transgenic plants under control conditions. As expected, an obvious increase in the free proline content was observed under cold stress in both WT and transgenic plants. However, the *OsPCF6* and *OsTCP21* RNAi lines accumulated more free proline than WT under cold stress (Fig. 8B). These results indicate that *OsPCF6* and *OsTCP21* negatively regulated plant cold tolerance, perhaps through modulating the accumulation of free proline under cold stress.

Reactive oxygen species (ROS) generation is usually observed in damaged cells during cold stress. Considering the damage caused by ROS, we evaluated the ROS generation in leaves of WT, OE and Ri seedlings which were exposed to cold stress. H_2_O_2_ formation was visualized by polymerization with 3, 3′ -diaminobenzidine (DAB). As shown in Fig. 8C, in the OE seedling leaves, H_2_O_2_ accumulation was observed as dark brown deposits, indicating that OE plants exhibited the weakest ability to eliminate ROS. The *OsPCF6* and *OsTCP21* Ri plants had the strongest ability to eliminate ROS, and thus, displayed the lightest color. These results suggested that *OsPCF6* and *OsTCP21* regulated plant cold tolerance by mediating ROS generation or scavenging.

**Figure 9:**
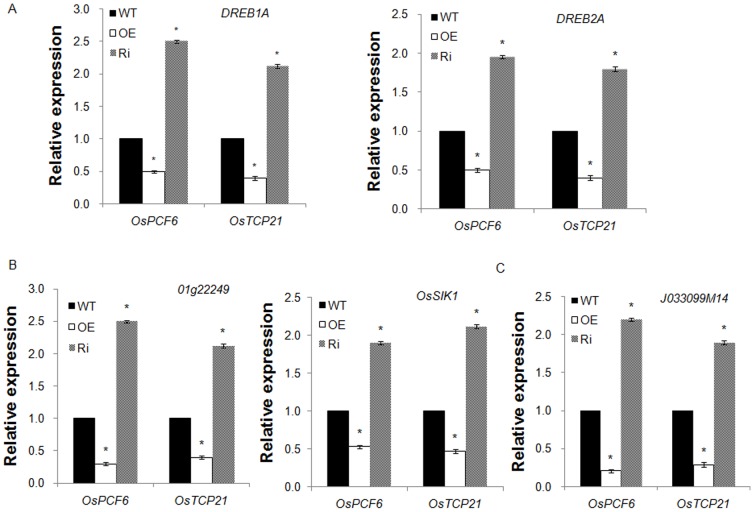
Expression levels of some cold-responsive genes in wild-type plants and *OsPCF6* and *OsTCP21* transgenic plants. (A) Expression levels of *DREB1A* and *DREB2A* gene in transgenic lines and wild-type plants. (B) Expression levels of ROS scavenging genes (*Os01g22249* and *OsSIK1*) in transgenic lines and wild-type plants. (C) Expression levels of putative proline synthase gene (*J033099M14*) in transgenic lines and wild-type plants. Data are the mean ± SE of three biological replicates. Asterisks indicate statistically significant differences (*P<0.05) between WT and transgenic lines.

To further elucidate the mechanism by which *OsPCF6-* and *OsTCP21-*RNAi plants enhanced cold tolerance the effects of cold stress on expression of genes responsible for proline biosynthesis and H_2_O_2_ scavenging were investigated. As shown in Figure 9, treatment with cold stress led to a great increase in transcripts of *DREB1A* and *DREB2A* and H_2_O_2_ scavenging (*Os01g22249* and *OsSIK1*) and proline biosynthesis genes (D-1-pyrroline-5-carboxylate synthase genes, *J033099M14*) in the *OsPCF6-* and *OsTCP21-*RNAi lines than wild-type and overexpression lines was observed in response to cold stress.

### *OsPCF6* and *OsTCP21* proteins are located in the nuclei and the spatial-temporal expression profiles of *Osa-miR319b* and *OsPCF6* and *OsTCP21*

**Figure 10:**
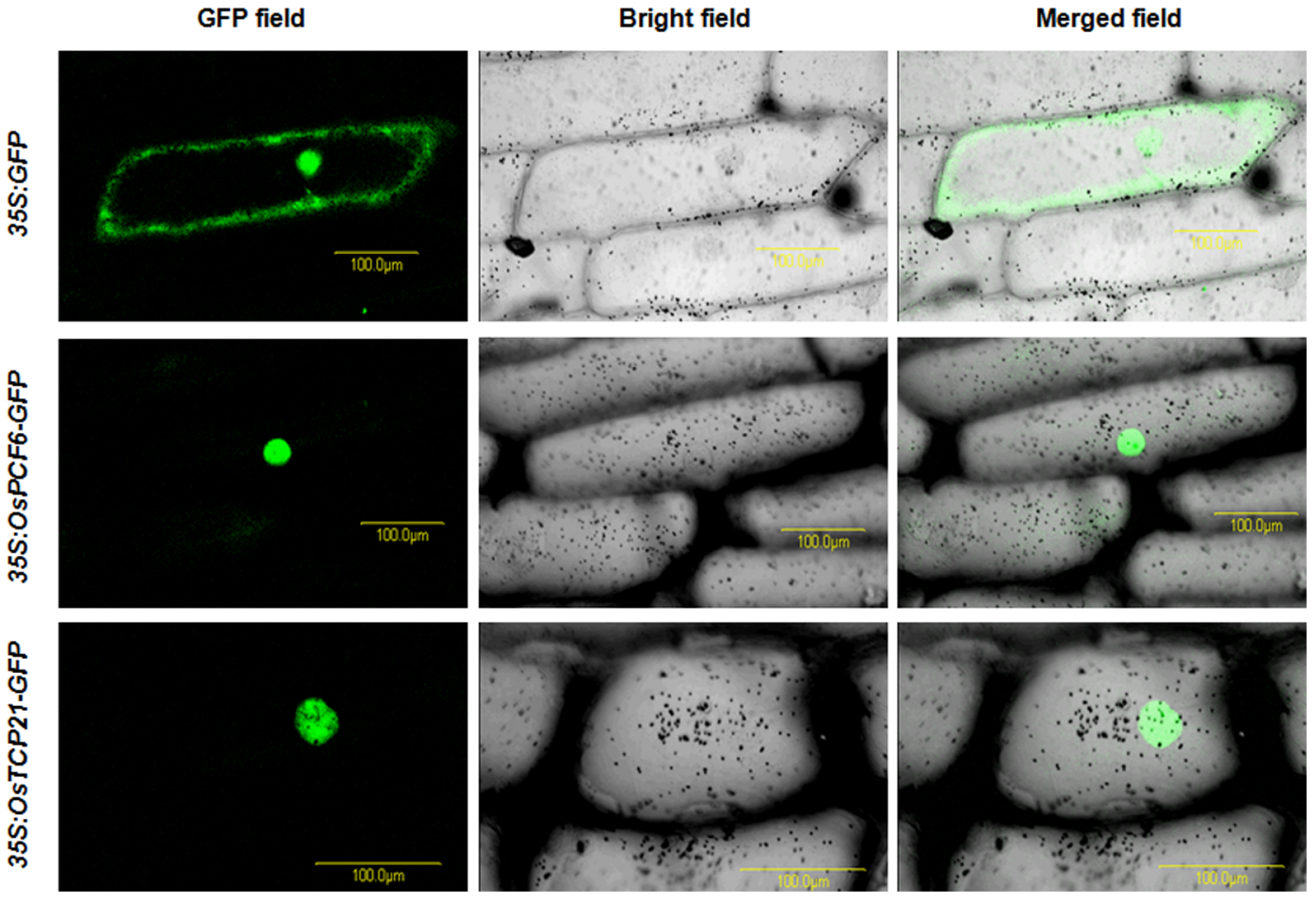
OsPCF6-GFP and OsTCP21-GFP proteins were located in the nuclei. Confocal images of onion epidermis cells under the GFP channel showing the constitutive localization of GFP and the nuclear localization of OsPCF6- and OsTCP21-GFP fusions driven by the cauliflower mosaic virus 35S promoter. Onion epidermal peels were bombarded with DNA-coated gold particles and GFP expression was visualized 24 h later.

As transcription factors, *OsPCF6* and *OsTCP21* are supposed to be located in the nuclei. To examine the subcellular localization of *OsPCF6* and *OsTCP21* proteins, the full-length coding regions of *OsPCF6* and *OsTCP21* were fused in-frame to the 5′-terminus of the GFP reporter under the control of the CaMV35S promoter. The OsPCF6-GFP and OsTCP21-GFP recombinant constructs and GFP alone were introduced into onion (*Allium cepa*) epidermal cells by particle bombardment. An examination of protein fluorescence by confocal laser-scanning microscopy revealed that the OsPCF6-GFP and OsTCP21-GFP fusion proteins were specifically localized in the nuclei, whereas GFP alone showed a ubiquitous distribution in the cell (Fig. 10).

Previous studies showed higher expression of *Osa-miR319b* in the young panicle and culm, and lower expression in root, mature panicle, and leaf. Also, *OsTCP21* was mainly detected in later panicle stages, ovaries and stigma, whereas, *OsPCF6* showed higher accumulation in shoot apical meristem, embryo and endosperm [19], [59]. *Osa-miR319b* was not inversely correlated with the *OsPCF6* and *OsTCP21* in spatio-temporal expression specificity, suggesting that there exists a complex relationship between *Osa-miR319b* and its above target genes in rice.

## Discussion

It has been suggested that miRNA play essential roles in regulation of gene expression for normal growth and development and adaptation to environmental stresses [33]. In previous study, ectopic expression of miR319 induced larger leaf blades and continuous growth of leaf margins in snapdragon, Arabidopsis, tomato and other species though each with its distinct leaf form [20], [22]. In our study, we observed that overexpressing *Osa-miR319b* could result in wider leaf and delayed development in seeding stage of rice (Fig. 2). This result is generally similar to the larger leaves phenotype observed in transgenic dicotyledonous plants with miR319 overexpression [18], [20], [22]. Yang (2013) showed that wider leaves of overexpressing *Osa-miR319b* plants are not due to change in their cell size but to their increase cell number reflected mainly by increased leaf small veins. However, how affected the leaf width and delayed development of *Osa-miR319b* in our further study.

Numerous cold-regulated miRNAs have been identified in *Arabidopsis* [34], [35] and *rice* [13]. Previous studies suggested that *miR319b* responded to cold stress and that *TCP* transcription factors were the predicted targets of *miR319b* [13], [25], [19]. Transcription factors up-regulated by cold stress contained the TCP-domain families [36], leading us to conduct further experiments to characterize the function of *miR319b* and *TCP* in plant cold stress response. Both our and Yang study demonstrated that overexpressing *Osa-miR319b* led to enhanced cold tolerance (4°C) in transgenic rice seeding. Furthermore, cold stress *osa-miR319b* expression was down-regulated while the expression of miR319-targeted genes was up-regulated. The study by Yang observed that down-regulating the expression of either of the two miR319-targeted genes, *OsPCF5* and *OsPCF6*, in RNAi plants also resulted in enhanced cold tolerance. In this study, we focused on another two targets of *Osa-miR319b*, *OsPCF6* and *OsTCP21*. We found that *OsPCF6* and *OsTCP21* expression was largely greatly by cold stress, and the degree of induction was obviously repressed in *Osa-miR319b* transgenic lines. Consistent with previous study we also demonstrated that overexpression or repression *OsPCF6* and *OsTCP21* significantly decreased or increased plant cold torlerance. Moreover, we also reported that *OsPCF6* and *OsTCP21* negatively regulated ROS generation or scavenging under cold stress.

To evaluate the role of *Osa-miR319b* in plant tolerance to cold stress, in this study, we generated transgenic plants over-expressing *Osa-miR319b* by Agrobacterium-mediated transformation (Fig. 1B, C). One important finding was that *Osa-miR319b*-overexpressing plants exhibited higher survival rate than WT plants when exposed to cold stress (Fig. 3B). Therefore, *Osa-miR319b* possibly participates in and responds to cold stress, and over-expression of *Osa-miR319b* enhances plant tolerance to cold stress.

To elucidate the potential mechanisms for the enhanced cold tolerance of *Osa-miR319b* OE plants, an analysis of the proline content was conducted to monitor changes in physiological processes associated with plant cold stress tolerance. The accumulation of free proline [37], [38] is a common phenomenon in response to cold stress. The accumulated free proline to facilitates osmoregulation and protect plants from the dehydration that results from cold stress by reducing the water potential of plant cells. This study found a greater accumulation of free proline in the *Osa-miR319b* OE plants, which may partially account for the higher tolerance of *Osa-miR319b* OE plants to cold stress (Fig. 3C).

In addition to the *Osa-miR319b* OE plants can accumulation of free proline content, we also found that overexpression of *Osa-miR319b* led to higher expression levels of cold stress related genes, such as Hsieh showed that transgenic expression of the transcriptional activator, CRT/DRE-binding factor 1 (CBF1), enhanced the cold tolerance of tomato plants(Fig. 4). *DREB1/2* have been shown to play irreplaceable roles in cold stress [39], [40]. In rice, *OsDREB1A*, *OsDREB1B*, *OsDREB1C* and *OsDREB2A* have been studied in depth [41]. We demonstrated up-regulated expression of *DREB1* genes in OE plants under cold stress compared with wild-type plants. *OsCPT* is the deduced target gene of the *DREB* pathway via the *DRE/CRT* cis-element [42]. In our study, *OsCPT* displayed down-regulated expression in *Osa-miR319b* OE plants under cold stress. These results demonstrated that *Osa-miR319b* played an important role in cold tolerance by regulating a number of cold stress related genes.

In rice, miR319 was predicted to target five TCP genes, *OsPCF5*, *OsPCF6*, *OsPCF7*, *OsPCF8*, and *OsTCP21*. The abundance of the five TCP genes in different tissues of rice. As Yang showed that the highest expression of *OsPCF5* and *OsPCF8* was detected in the leaf and root, and that of *OsPCF7* and *OsTCP21* in the leaf and young panicle, *OsPCF6* was highly expressed in the young and mature panicle. Moreover, the miR319-targeted genes were up-regulated by low temperature treatments, the inverse-correlated with *Osa-miR319b*. For example, down-regulation of either *OsPCF5* or *OsPCF8* significantly improved the cold tolerance of transgenic seedlings cold tolerance [19], in sugarcane PCF6, which was identified as *miR319b* target gene by 5′RACE was induced by cold tolerance [32]. The expression patterns of *OsPCF6* and *OsTCP21* suggested potential roles in cold stress response (Fig. 5). The *OsPCF6/OsTCP21* Ri and OE plants showed enhanced and/or decreased tolerance to cold stress than WT, respectively (Fig. 7). One important finding was that RNAi plants exhibited a higher survival rate than WT plants (Fig. 8A). The cold stress phenotype of *OsPCF6* and *OsTCP21* OE plants counters the phenotype of the *Osa-miR319b*-overexpressing plants, while the RNAi plant's phenotypes are in keeping with those of the *Osa-miR319b*-overexpressing plants (compare Fig. 3A and 7). The phenotypic analysis indicated that both *OsPCF6* and *OsTCP21* were involved in cold responses, and that they may be regulated by *Osa-miR319b*.

To elucidate the mechanisms responsible for the enhanced tolerance of Ri lines, we analyzed the levels of free proline in *OsPCF6/OsTCP21* Ri and OE seedlings. The levels of free proline has been increased in Ri lines under cold stress (Fig. 8B), which may conferring the cold tolerance. Cold stress also causes increased levels of ROS, the elevated concentrations of ROS can damage cellular structures and macromolecules, leading to cell death [43], [44]. An increased ROS-scavenging ability might be beneficial to plant cold stress tolerance. As Huang reported, cold tolerance was enhanced when *OsZFP245* up-regulated the activities of ROS-scavenging enzymes [45]. The less accumulation of H_2_O_2_ under cold stress may result from an enhanced capacity for scavenging ROS, and the finding that the Ri lines had the lightest colors than in the wild-type and OE plants (Fig. 8C). The greater tolerance of RNAi plants to cold stress found in this study may be accounted for, at least in part, by mitigating oxidative damage due to suppression of ROS production.

To further elucidate the mechanism underlying the greater accumulation of ROS and proline in the *OsPCF6*/*OsTCP21*-over-expressing plants under cold stress, this study examined the changes in proline synthase (D-1-pyrroline-5-carboxylate synthase genes, *J033099M14*) and ROS scavenging genes (*Os01g22249* and *OsSIK1*) in *OsPCF6*- and *OsTCP21*-overexpressing, Ri, and wild-type plants in response to cold stress at the transcriptional level. The present results demonst rate that express ion of proline synthase and ROS scavenging genes was higher in *OsPCF6*- and *OsTCP21*-Ri plants than in wild-type and *OsPCF6-* and *OsTCP21*-OE plants under cold stress, suggesting that the higher proline content s and lower ROS contents in *OsPCF6-* and *OsTCP21*-Ri plants are likely to result from the greater up-regulation of these genes under cold stress (Fig. 9B,C). These results indicate that *OsPCF6* and *OsTCP21*play an important role in cold tolerance in rice by regulating proline contents and ROS scavenging.

**Figure 11:**
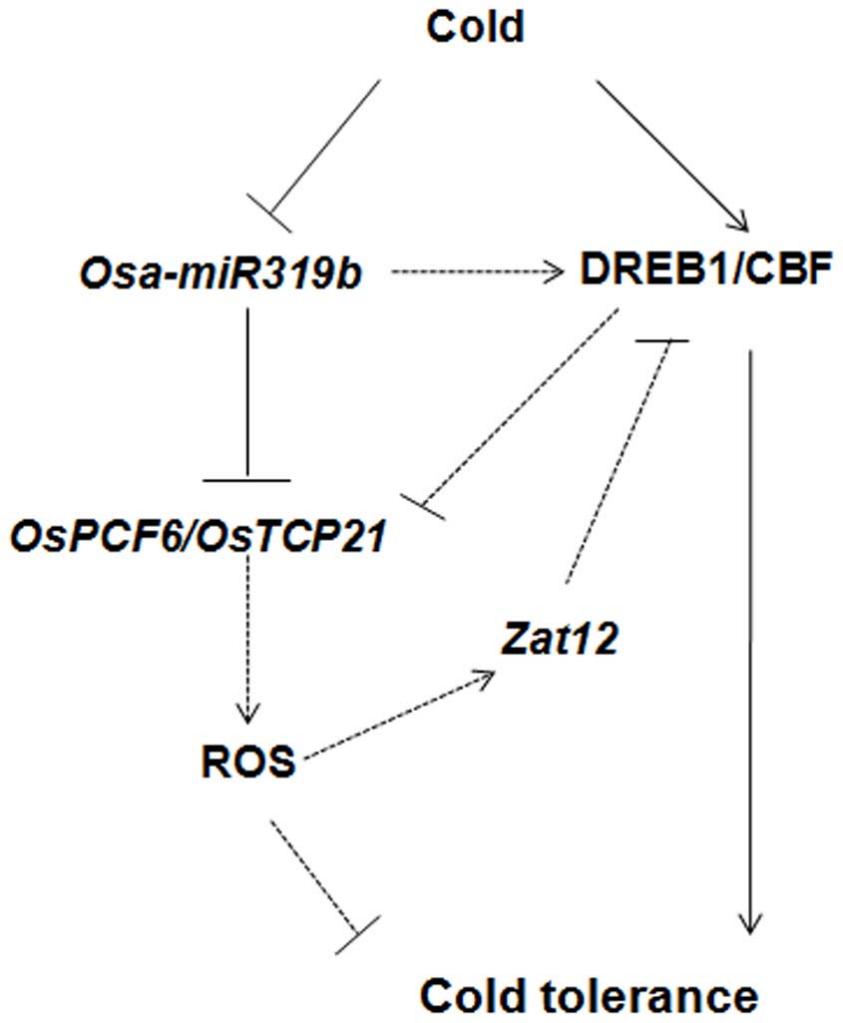
Possible molecular mechanism of cold stress in rice. Under cold stress *Osa-miR319b* is down-regulated, leading to the repression of its targets and possibly the up-regulation of ROS (arrow with dashed line), which in turn inhibits cold tolerance. Under cold stress *DREB1/CBF* is up-regulated, and *Zat12* down-regulated CBF transcript expression, and account of ROS up-regulated *Zat12* transcript expression which in turn increases cold tolerance. However, it is possible that *Osa-miR319b* induces the expression of *DREB1/CBF* (arrow with dashed line and question mark).

Based on this study, a hypothetical mechanistic model of the regulation by *Osa-miR319b* and its targets in rice has been proposed (Fig. 11), but how *Osa-miR319b* and its targets participate in cold stress remains to be determined. Exposure of rice to cold induces decreased *Osa-miR319b* expression, which regulates the expression of the proposed target genes *OsPCF6* and *OsTCP21*. Hsieh showed that transgenic expression of the transcriptional activator, CRT/DRE-binding factor 1 (CBF1), enhanced the cold tolerance of tomato plants [44], [46]. *Zat12*, an ROS-response zinc finger protein was shown to regulate cold-induced genes. Microarray analysis demonstrated that cold-responsive genes were upregulated by overexpression of *Zat12* [47], and *Zat12* downregulated CBF transcript expression suggesting a role for *Zat12* in suppressing the CBF cold-response pathway. These studies demonstrate a close link between CBF, ROS signaling, and the cold stress response [4]. Up-regulated *Osa-miR319b* and down-regulated *OsPCF6* and *OsTCP21* can increase the account of CBF (Fig. 4, 9A), but *Zat12* down-regulated CBF transcript expression, ROS up-regulated *Zat12* transcript expression suggesting that decrease amount of ROS improve cold resistance. This result suggested that up-regulated *Osa-miR319b* and down-regulated *OsPCF6* and *OsTCP21* enhanced cold tolerance possibly through decreased levels of ROS.

Cold temperatures negatively affect plant growth and development by causing tissue injury and delaying growth [48], and thereby significantly restrict the spatial distribution of plants and the productivity of forests. A plant's ability to withstand cold temperatures depends on its ability to regulate gene expression that modifies their physiology, metabolism, and growth [49]. Diverse plant species endure cold stress to varying degrees, and *miR319* was up-regulated in two varieties of sugarcane [32] and down-regulated in the two major subspecies of rice [13] exposed to cold stress. The ability to tolerate low temperatures is a major distinguishing factor in classifying the two major rice subspecies, *japonica* and *indica* [50]. Andaya reported that the *japonicas* are more cold tolerant than the *indicas* [51]. The expression of *Osa-miR319b* in *japonica* rice was reduced beginning at 3 h after exposed to cold, and there was almost no expression at 24 h [13]. However, in *indica* rice, the expression of *Osa-miR319b* increased between 12 and 24 h after exposed to cold [19]. The result show differences in *Osa-miR319b* regulation among different rice genotypes contrasting in tolerance to cold. These results suggest that differences in the intensity of the regulation of *Osa-miR319b* could be tested as a marker for selecting cold-tolerant rice cultivars.

In summary, this study indicates that *Osa-miR319b* functions as a positive regulator to mediate the tolerance of rice seedlings to cold stress. The overexpression of *Osa-miR319b* and RNAi of *OsPCF6* and *OsTCP21* led to a greater accumulation of osmolytes, such as free proline, and *DREB1/CBF* proteins in rice, and suppressed the accumulation of ROS under conditions of cold stress through down-regulating *OsPCF6* and *OsTCP21*, the targets of *Osa-miR319b*. The up-regulation of *Osa-miR319b* or RNAi of *OsPCF6* and *OsTCP21* may allow rice plants to effectively osmoregulate by accumulating free proline and to minimize oxidative damage in plants under abiotic stress. *OsPCF6* and *OsTCP21* are a kind of transcription factors [25], located in the nuclei (Fig. 10) and the function under cold stress have been characterized by generating OE and RNAi transgenic rice plants. More importantly, overexpression of *Osa-miR319b* and the OE and RNAi of *OsPCF6* and *OsTCP21* in rice seedlings did not affect their phenotypes under control conditions. Therefore, *Osa-miR319b*, *OsPCF6* and *OsTCP21* provide a promising tool for improving the tolerance of rice to abiotic stress in general and to cold stress in particular.
